# Outcomes of bidirectional Glenn surgery done without prior cardiac catheterization

**DOI:** 10.1186/s43044-022-00296-4

**Published:** 2022-08-04

**Authors:** Ahmad Azhar, Rehan Eid, Ahmed Elakaby, Mohamed Abdelsalam, Jameel Al-Ata, Naif Alkhushi, Saud Bahaidarah, Zaher Zaher, Khadijah Maghrabi, Nada Noaman, Gaser Abdelmohsen

**Affiliations:** 1 Pediatric Cardiology Division, Department of Pediatrics, King Abdulaziz University, P.O Box 80215, Jeddah, 21589 Saudi Arabia; 2 Paediatric Department, Al-Azhar University, Cairo, Egypt; 3 Cardiology Department, Benha University, Benha, Egypt; 4 Department of Anesthesia and Critical Care, King Abdulaziz University, P.O Box 80215, Jeddah, 21589 Saudi Arabia; 5 Pediatric Cardiology Division, Department of Pediatrics, Cairo University, Cairo, 11562 Egypt

**Keywords:** Cavopulmonary shunt, Bidirectional Glenn, Cardiac catheterization, Restrictive pulmonary flow

## Abstract

**Background:**

Cardiac catheterization is usually done routinely in patients with univentricular hearts before palliative Bidirectional Glenn (BDG) surgery. The objective of this study was to compare the outcomes of patients with physiological univentricular hearts and restrictive pulmonary flow that did not undergo routine cardiac catheterization before BDG with the patients that did have cardiac catheterization done. We retrospectively reviewed the data of all patients with single ventricle physiology and restrictive pulmonary blood flow who underwent BDG surgery from January 2016 till December 2020. Patients were divided into two groups: the catheterization and the non-catheterization groups.

**Results:**

Out of 93 patients, 25 (27%) underwent BDG surgery without prior cardiac catheterization. The median age of patients was ten months, interquartile range (IQR) was 5–18 months. Tricuspid atresia represented 36% of the non-catheterization group, while unbalanced atrioventricular septal defect and hypoplastic left heart syndrome represented 19% and 17.6% of the catheterization group. No patients in the catheterization group were excluded from further BDG surgery based on the catheterization data. Moreover, no significant differences were found between the patients' groups regarding the length of hospital stay, length of intensive care unit stay, postoperative oxygen saturation, or survival (*P* = 0.266, *P* = 0.763, *P* = 0.543, *P* = 0456).

**Conclusions:**

Although pre-BDG cardiac catheterization is the routine and standard practice, in certain situations, some patients with single ventricle physiology and restrictive pulmonary blood flow may go directly to BDG without cardiac catheterization if noninvasive imaging is satisfactory on a case-by-case basis and according to center experience. Pre-BDG catheterization could be reserved for patients with limited echocardiographic studies, high-risk patients, or those indicated for catheter intervention before BDG surgery.

## Background

Patients with single ventricle physiology typically need multiple palliative procedures to secure pulmonary blood flow and decrease the load on the dominant ventricle. Partial cavopulmonary connection (PCPC) or bidirectional Glenn (BDG) surgery is usually performed for these patients in infancy as a second stage palliative surgery before Fontan palliation. In BDG, the superior caval vein is anastomosed surgically to the right pulmonary artery [1]. In routine practice, cardiac catheterization is usually performed before BDG. Pre-BDG catheterization can provide essential hemodynamic and anatomical information for the surgical procedure. It can also give an idea about the operative risk and postoperative prognosis.

The main drawback of pre-BDG catheterization is the risk of radiation exposure, the complications related to the catheterization procedure, and the need for hospital admission. In clinical practice, and in addition to previously published reports, very rarely were patients excluded from further Glenn surgery based on cardiac catheterization data [2–5]. In the last five years, we noticed that none of the patients with univentricular heart physiology and restrictive pulmonary flow that underwent pre-BDG catheterization were excluded from further surgery. Additionally, some patients with restrictive pulmonary flow underwent BDG surgery without prior cardiac catheterization. This finding raises a few crucial questions that we aim to answer. Is pre-BDG cardiac catheterization essential for evaluating all patients with limited (restrictive) pulmonary blood flow? Does it really affect the outcome of these patients? The main objective of this study was to compare the outcome of patients with physiological univentricular hearts and restrictive pulmonary blood flow that did not perform pre-BDG cardiac catheterization with the outcome of patients who underwent pre-BDG catheterization.

## Methods

According to hospital policy, informed consents were obtained from all patients' legal guardians prospectively on hospital admission regarding possible recruitment of their data in any future research. The local ethical committee approved the retrospective study. In this cohort, all patients with physiological univentricular hearts and restrictive pulmonary blood flow that underwent palliative BDG surgery were retrospectively included in the study. We considered patients to have restrictive pulmonary blood flow if the pressure gradient (PG) was > 45 mmHg during echocardiography across the following structures: pulmonary valve, subpulmonic infundibulum, pulmonary artery band, branch pulmonary arteries, Sano shunt after Norwood surgery, Modified Blalock–Taussig–Thomas shunt (MBTTS), patent ductus arteriosus (PDA) stent, or ventricular septal defect if pulmonary artery arises from the rudimentary ventricle (as in cases with tricuspid atresia with normally related vessels) [3].

In contrast, patients with increased pulmonary blood flow before BDG surgery were excluded. We also excluded all patients with clinical or radiological criteria that could suggest increased pulmonary blood flow, such as signs or symptoms of heart failure, oxygen saturation above 80%, and increased vascular markings on chest X-ray. Data were collected from the medical records stored in the hospital computer system.

## Statistical analysis

Statistical analysis was performed using IBM SPSS Statistics for Windows, version 26.0 (IBM Corp., Armonk, NY, USA). Categorical variables were expressed as numbers and percentages, while numeric variables were presented as medians and interquartile ranges (25th–75th percentiles). Comparisons between groups were tested using the χ2 and Mann–Whitney U tests for categorical and numeric variables, respectively. Statistical significance was considered if the *P*-value was < 0.05.

## Results

### Clinical characteristics

The total number of patients was 93 patients; 25 patients (27%) of them underwent BDG surgery without prior cardiac catheterization. Although the patients in the non-catheterization group were relatively younger and had a lower preoperative oxygen saturation than the catheterization group, these differences did not reach a statistically significant level (*P* = 0.06, 0.07, respectively). A large proportion of the non-catheterization group had tricuspid atresia (36%) compared to the catheterization group, which comprised a large proportion of unbalanced atrioventricular septal defect (19%) and hypoplastic left heart syndrome (17.6%) as illustrated in Table 1. Regarding prior surgical procedures/interventional catheterization, most of the non-catheterization group did not perform previous intervention compared to the catheterization group (84% versus 31%, *P* = 0.002). Table 1 shows the clinical characteristics of the patients' groups.

**Table 1:** Clinical characteristics of patients' groups

	Non-catheterization group (*n* = 25)	Catheterization group (*n* = 68)	*P* value
Age, months	7 (3–12)	10.5 (5–23.5)	0.060
Weight, Kg	6 (4.8–8.7)	7.7 (6–9.9)	0.085
Follow-up period, months	18 (1.5–40)	32 (13–57)	0.025
Male/female, *n* (%)	15 (60)/10 (40)	41 (60.3)/27 (39.7)	0.980
Oxygen saturation, %	70 (55–75)	72 (68–77)	0.077
Diagnosis, *n* (%)			0.126
HLHS/Variant	2 (8)	12 (17.6)	
Tricuspid atresia	9 (36)	10 (14.7)	
DILV	1 (4)	10 (14.7)	
Unbalanced AVSD	1 (4)	13 (19.1)	
DORV, non-committed VSD	6 (24)	11 (16.2)	
PA/IVS	2 (8)	4 (5.9)	
Others	4 (16)	8 (11.8)	
Dominant ventricle			0.462
RV dominance	4 (16)	17 (25)	
LV dominance	11 (44)	32 (47)	
Biventricular/indeterminate	10 (40)	19 (28)	
Prior intervention, *n* (%)			0.002
No prior intervention	21 (84)	21 (31)	
PDA stent	3 (12)	22 (32.4)	
Norwood	1 (4)	16 (32.5)	
MBTTS	0	3 (4.4)	
RVOT stent	0	1 (1.5)	
Pulmonary artery banding	0	5 (7.4)	

*HLHS* hypoplastic left heart syndrome, *DILV* Double inlet left ventricle, *AVSD* atrioventricular septal defect, *DORV* Double outlet right ventricle, *VSD* ventricular septal defect, *PA/IVS* pulmonary atresia with intact ventricular septum, *PDA* patent ductus arteriosus, *MBTTS* Modified Blalock–Taussig–Thomas shunt., *RVOT* right ventricle outflow tract

### Echocardiography and cardiac catheterization

Echocardiography plays an essential role in the preoperative evaluation of patients with univentricular heart physiology before BDG. It can provide most of the information needed before BDG surgery. In this cohort, echocardiography identified five patients with significant atrioventricular regurgitation who underwent valve repair during the BDG procedure, 12 patients with left SVC who underwent bilateral BDG, and 24 with pulmonary artery stenosis underwent pulmonary artery plasty. Echocardiography is limited in evaluating distal pulmonary arteries and aortopulmonary collateral arteries (APCs). These vessels are better delineated by selective angiography during cardiac catheterization or by another imaging modality like multidetector computed tomography (MDCT). Pre-BDG cardiac catheterization identified seven patients with APCs; all were nonsignificant and did not require percutaneous closure before or after BDG surgery. Echocardiography showed aortic arch obstruction (pressure gradient > 10 mmHg) in two patients with hypoplastic left heart syndrome (HLHS) after the Norwood procedure who underwent successful balloon dilatation. All patients who underwent cardiac catheterization had favorable hemodynamics: median ventricular end-diastolic pressure (EDP) was 12 mmHg, and median mean pulmonary artery pressure (PAP) was 16 mmHg. In contrast, median indexed pulmonary vascular resistance (PVRi) was 1.4 Wood unit.m^2^. None of our patients underwent MDCT, cardiac magnetic resonance imaging (MRI), or percutaneous angioplasty of the pulmonary arteries. Table 2 shows the echocardiographic and catheterization data of the patient groups.

**Table 2:** Echocardiographic, catheterization and surgical data of patients' groups

	Non-catheterization group *N* = 25	Catheterization group *N* = 68	*P*-value
*Echocardiographic data*			
Moderate AVV regurgitation, *n* (%)	1 (4)	4 (5.9)	0.750
Branch pulmonary artery stenosis, *n* (%)	5 (20)	19 (28)	0.787
Aortic arch obstruction, *n* (%)	–	2 (2.9)	
Persistent LSVC, *n* (%)	3 (12)	9 (13.2)	0.875
Significant aortic/Neo aortic regurgitation	–	–	
*Catheterization data*			
Hemodynamics			
EDP, mmHg		12 (9–15)	
Mean PAP, mmHg		16 (13–19)	
PVRi, WU m^2^		1.4 (0.9–2.2)	
Angiography, *n* (%)			
No/mild RPA stenosis		56 (82.3)	
Moderate RPA stenosis		8 (11.8)	
Severe RPA stenosis		4 (5.9)	
No/mild LPA stenosis		59 (86.8)	
Moderate LPA stenosis		5 (7.4)	
Severe LPA stenosis		2 (3)	
Aortic arch obstruction		2 (2.9)	
Persistent LSVC		9 (13.2)	
APCs		7 (10.3)	
Preoperative catheter interventions		2 (2.9)	
*Surgical data*			
Bypass time, minute	44 (40–65)	46 (40–60)	0.903
Unilateral/Bilateral Glenn, *n* (%)	22 (88)/3 (12)	59 (86.8)/9 (13.2)	0.875
Antegrade pulmonary flow, *n* (%)	9 (36)	13 (19)	0.089
Additional surgical procedure			
Pulmonary artery plasty *n* (%)	5 (20)	21 (31)	0.787
Atrioventricular valve plasty *n* (%)	1 (4)	4 (5.9)	0.750
Atrial septectomy *n* (%)	12 (48)	19 (27.9)	0.055
Pacemaker insertion (congenital heart block) *n* (%)	–	1 (1.5)	
Operative complications *n* (%)	–	–	

*AVV* atrioventricular valve, *LSVC* left superior vena cava, *EDP* end-diastolic pressure, *PAP* pulmonary artery pressure, *PVRi* indexed pulmonary vascular resistance, *RPA* right pulmonary artery, *LPA* left pulmonary artery, *APCs* aortopulmonary collaterals

### Surgical procedures

All the patients underwent median sternotomy with cardiopulmonary bypass. Twelve patients had bilateral SVC and underwent bilateral BDG surgery. Twenty-six patients performed additional pulmonary artery plasty, 31 performed atrial septectomy, 5 had atrioventricular valve repair for atrioventricular valve regurgitation, and only one had double inlet ventricle associated with congenital heart block underwent epicardial permanent dual-chamber pacemaker (DDD). No surgical complications were reported in this cohort. There was no statistically significant difference between the non-catheterization group and the catheterization group regarding the surgical procedure, as illustrated in Table 2.

### Postoperative course and outcome

All patients had a central venous line inserted in the internal jugular vein, which measured postoperative mean pulmonary artery pressure. The median mean PAP was 14 mmHg in the non-catheterization group compared to 13 mmHg in the catheterization group, with no significant difference between both groups (Table 3).

**Table 3:** Outcome of patients' groups

	Non-catheterization group *N* = 25	Catheterization group *N* = 68	*P*-value
Postoperative			
CVP/mean PAP, mmHg	14 (18–21)	13 (18–20)	0.433
Postoperative Glenn obstruction, *n* (%)	–	1 (1.5)	
Postoperative catheterization, *n* (%)	–	1 (1.5)	
Low COP, *n* (%)	1 (4)	1 (1.5)	0.456
Subdural hematoma, *n* (%)	–	1 (1.5)	
Glenn obstruction, *n* (%)	–	1 (1.5)	
Diaphragmatic paralysis, *n* (%)	–	1 (1.5)	
Chylothorax, *n* (%)	4 (16)	3 (4.4)	0.060
Outcome			
Hospital length of stay, days	8 (7–10)	8 (6–11)	0.266
Oxygen saturation on discharge, %	84 (80–88)	83 (80–86)	0.543
ICU length of stay, days	5 (3.5–7)	5 (3–7)	0.763
Mortality, *n* (%)	1 (4)	1 (1.5)	0.456
Follow-up			0.118
Fontan	6 (24)	26 (38.2)	
Waiting Fontan	10 (40)	34 (50)	
Lost follow-up	7 (28)	6 (8.8)	
One and half repair	1 (4)	1 (1.5)	

*CVP* central venous pressure, *PAP* pulmonary artery pressure, *COP* cardiac output, *ICU* intensive care unit

In this cohort, one patient had postoperative Glenn obstruction managed by percutaneous balloon angioplasty. Two patients had low cardiac output and represented the only mortalities in the study. One patient had left phrenic palsy that required diaphragmatic plication, while seven patients had chylothorax (that was managed conservatively). There were no significant differences between the patients' groups regarding chylothorax, hospital stay, intensive care unit stay, postoperative oxygen saturation, or survival, as illustrated in Table 3.

## Discussion

Patients with univentricular hearts are more vulnerable to morbidity and mortality than those with biventricular hearts [1]. Identification of patients at risk before palliative BDG and Fontan shunts is essential. Poor ventricular function, significant atrioventricular valve regurgitation (AVVR)/stenosis, aortic coarctation/obstruction, collaterals of hemodynamic significance, elevated pulmonary vascular resistance > 3 Wood units m^2^, distortion/ hypoplasia of pulmonary arteries are identifiable risk factors associated with significant morbidity and mortality in patients with univentricular hearts [6–9]. In most cases, echocardiography can provide anatomical and functional data required before BDG surgery. Echocardiography has limitations in the evaluation of distal pulmonary arteries and APCs in addition to its dependence on the acoustic windows that may not be optimal, especially in chest deformities after previous cardiac surgeries and hyperinflated lungs [4, 10]. In this cohort, echocardiography was the only diagnostic modality used for preoperative evaluation in the non-catheterization group. Echocardiographic studies in the non-catheterization group were sufficient and clear enough that no other diagnostic modalities like MDCT or cardiac magnetic resonance imaging (MRI) were additionally performed. Moreover, all findings detected by echocardiography were found and fixed intraoperatively. Although echocardiography has a limitation in the evaluation of PVR or mean PAP in patients with univentricular heart (if pulmonary regurgitation Doppler tracing is inadequate), the clinical picture of these patients did not suggest elevated PVR. Hence, they underwent BDG without the need for cardiac catheterization.

Echocardiography can visualize the proximal pulmonary arteries with great clarity. In our study, echocardiography identified 19 patients with proximal pulmonary artery stenosis in the catheterization group out of 21 patients identified by cardiac catheterization with a detection rate of 90%. Although we did not opt to do it in our study, MDCT angiography is an excellent tool for evaluating peripheral pulmonary arteries and APCs. It is even superior to MRI for evaluating peripheral pulmonary arteries. It can be used as the second step for evaluating pulmonary arteries and APCs if echocardiography is inadequate without the need for cardiac catheterization [2, 11, 12].

APCs usually develop secondary to long-standing desaturation associated with decreased or progressively decreasing pulmonary blood flow. The development of APCs is a compensatory mechanism to increase pulmonary flow and oxygen saturation. After BDG surgery, with patient growth, the upper part of the body becomes less dominant, and the Glenn flow will decrease gradually, leading to desaturation, especially if the Fontan shunt is done late. APCs may complicate the postoperative course secondary to increased pulmonary flow and elevating pressure in pulmonary arteries leading to lung plethora, respiratory distress, increased chest tube drainage, and pleural effusions. Large APCs of hemodynamic significance need to be closed immediately before Glenn surgery or postoperatively. Triedman et al. noticed an increased incidence of APCs in patients after Glenn surgery compared to the Fontan procedure [13]. In this study, seven patients had APCs after cardiac catheterization; all were nonsignificant, and none required preoperative or postoperative percutaneous closure. Echocardiography has a limited ability for visualization of APCs compared to MDCT. MDCT could be a second diagnostic modality for evaluating distal PAs, aortic arch, and APCs. MDCT angiography is less invasive, less time-consuming, and has minimal ionizing radiation with newer techniques [11]. Brawn et al. showed in their cohort that in patients who underwent just routine cardiac catheterization, no additional information or interventions were added by cardiac catheterization [14]. Although the noninvasive imaging alone cannot totally replace the diagnostic cardiac catheterization before BDG, in certain circumstances, patients may get the benefit of doing the surgery without delay, especially in developing countries with limited resources and overloaded cardiac catheterization laboratories with long waiting lists according to center experience. The favorable conditions that may encourage to go directly to BDG surgery *with caution* based on a case-by-case discussion are the following:

1. Patients have no symptoms or signs of heart failure.
2. Pulmonary and systemic venous circulations are not obstructed as in cases with restrictive interatrial communication and atresia of one of the atrioventricular valves.
3. Chest X-ray shows no signs of pulmonary venous congestion or plethora.
4. Echocardiography is of good quality to show detailed cardiac anatomy and function.

Although echocardiography cannot evaluate APCs, most patients with univentricular hearts usually do not develop significant APCs early in life if oxygen saturation is acceptable. These collaterals can be occluded after Glenn surgery if the patient has high chest tube drainage, heart failure symptoms, or before Fontan surgery. This may explain why none of our patients required APC closure before or after BDG surgery.

Interestingly, hemodynamic measurements in the catheterization group revealed favorable hemodynamics, and none of the patients was excluded from further Glenn surgery. These findings should raise the concern about the certainty of routine cardiac catheterization before every BDG surgery in patients with univentricular heart and restrictive pulmonary flow if noninvasive imaging modalities are reassuring and the patient is at low risk for BDG surgery.

In this cohort, there was no significant difference between the catheterization and the non-catheterization groups regarding postoperative course or outcome. The mean PAP measured through the central line inserted in IJV did not differ significantly between patient groups. We did not find a significant difference between the patient groups regarding chylothorax, hospital stay, intensive care unit stay, oxygen saturations at discharge, or survival.

Brown et al. did not find significant differences in short-term or long-term outcomes between patients with univentricular hearts who underwent routine pre-BDG catheterization and those who underwent only cardiac MRI [12]. We think that with time, the noninvasive imaging modalities will gradually replace diagnostic cardiac catheterization, especially with the rapid progress in artificial intelligence involved in the novel imaging tools [15]. Until then, the debate and argument will continue between who supports catheterization before every BDG surgery and who does not.

## Limitations

The main limitations of this cohort are it being a retrospective single-center cohort and the relatively small number of patients recruited, echocardiography was the only imaging modality, especially in the non-catheterization group, in addition to the heterogeneity of both groups regarding age, weight, and diagnosis so the selection bias could not be eliminated. More randomized control trials on larger scales of patients recruited from multiple cardiac centers are strongly recommended.

## Conclusions

Although pre-BDG cardiac catheterization is the routine practice and the standard of care, in certain situations, some patients with single ventricle physiology and restrictive pulmonary blood flow may go directly *with caution* to BDG if noninvasive imaging modalities are of good quality, reassuring, and patients are of low risk for BDG surgery. The decision should be made based on case-by-case discussion and center experience.

## Data Availability

All data were available at King Abdulaziz University Hospital.

## Abbreviations

APCs: Aortopulmonary collaterals
AVSD: Atrioventricular septal defect
AVV: Atrioventricular valve
AVVR: Atrioventricular valve regurgitation
BDG: Bidirectional Glenn
COP: Cardiac output
CVP: Central venous pressure
DILV: Double inlet left ventricle
EDP: End-diastolic pressure
HLHS: Hypoplastic left heart syndrome
ICU: Intensive care unit
MBTTS: Modified Blalock–Taussig–Thomas shunt
MDCT: Multidetector computed tomography
MRI: Magnetic resonance imaging
PA/IVS: Pulmonary atresia with intact ventricular septum
PAP: Pulmonary artery pressure
PCPC: Partial cavopulmonary connection
PDA: Patent ductus arteriosus
PG: Pressure gradient
LPA: Left pulmonary artery
PVRi: Indexed pulmonary vascular resistance
RPA: Right pulmonary artery
RVOT: Right ventricle outflow tract
SVC: Superior vena cava
VSD: Ventricular septal defect
WU: Wood unit
